# Pediatric Emergency Care Coordinator Presence and Pediatric Care Quality Measures

**DOI:** 10.1001/jamanetworkopen.2024.51111

**Published:** 2024-12-18

**Authors:** Margaret E. Samuels-Kalow, Rebecca E. Cash, Kenneth A. Michelson, Courtney Benjamin Wolk, Katherine E. Remick, Stephanie S. Loo, Maeve F. Swanton, Elizabeth R. Alpern, Kori S. Zachrison, Carlos A. Camargo

**Affiliations:** 1 Department of Emergency Medicine, Massachusetts General Hospital, Boston; 2 Division of Emergency Medicine, Ann & Robert H. Lurie Children’s Hospital of Chicago, Northwestern University Feinberg School of Medicine, Chicago, Illinois; 3 Department of Psychiatry, Perelman School of Medicine, University of Pennsylvania, Philadelphia; 4 Department of Pediatrics, Dell Medical School, The University of Texas at Austin, Austin

## Abstract

**Importance:**

Higher pediatric readiness has been associated with improved quality and outcomes of care for children. Pediatric emergency care coordinators (PECCs) are a component of pediatric readiness, but the specific association between PECCs and quality-of-care measures is undefined.

**Objective:**

To examine the association between PECC presence and emergency department (ED) performance as reflected by quality-of-care measures.

**Design, Setting, and Participants:**

This cohort study of ED patients 18 years or younger used data across 8 states, combining the 2019 National Emergency Department Inventory–USA, 2019 State Emergency Department Database and State Inpatient Database, 2020 Supplemental National Emergency Department Inventory PECC Survey, and the 2021 National Pediatric Readiness Project Survey. This analysis was conducted from February 15, 2023, to July 9, 2024.

**Exposure:**

Presence of a PECC.

**Main Outcomes and Measures:**

Hospitals were stratified by presence of pediatric resources (ie, pediatric intensive care and inpatient units), with exclusion of children’s hospitals and comparison between pediatric-resourced and non–pediatric-resourced (unable to admit children, no pediatric intensive care unit) hospitals. The 7 measures chosen were length of stay longer than 1 day for discharged patients, left against medical advice or without completing treatment, death in the ED, return visits within 3 days, return visits with admission within 3 days, use of chest radiography in patients with asthma, and use of head computed tomography for patients with head trauma. For each stratum, multilevel generalized linear models were constructed to examine the association between PECC presence and process and utilization measure performance, adjusted for patient-level factors (age, sex, race and ethnicity, insurance, and complex chronic conditions) and ED-level factors (visit volume, patient census, and case mix [race and ethnicity, insurance, and complex chronic conditions]).

**Results:**

There were 4 645 937 visits from pediatric patients (mean [SD] age, 7.8 [6.1] years; 51% male and 49% female) to 858 hospitals, including 849 non-freestanding pediatric hospitals, in the analytic sample. Highly resourced pediatric centers were most likely to have a PECC (52 of 59 [88%]) compared with moderately resourced (54 of 156 [35%]) and non–pediatric-resourced hospitals (66 of 519 [13%]). Among the 599 non–pediatric-resourced hospitals, PECC presence was associated with decreased rates of computed tomography in head trauma (adjusted odds ratio [AOR], 0.76; 95% CI, 0.66-0.87); in the pediatric-resourced hospitals, the AOR was 0.85 (95% CI, 0.73-1.00). For patients with asthma, PECC was associated with decreased chest radiography rates among pediatric-resourced hospitals (AOR, 0.77; 95% CI, 0.66-0.91) but not non–pediatric-resourced hospitals (AOR, 0.93; 95% CI, 0.78-1.12).

**Conclusions and Relevance:**

The presence of a PECC was not consistently associated with quality-of-care measures. The presence of a PECC was variably associated with performance on imaging utilization measures, suggesting a potential influence of PECCs on clinical care processes. Additional studies are needed to understand the role of PECCs in driving adherence to clinical care guidelines and improving quality and patient outcomes.

## Key Points

**Question:**

Is the presence of a pediatric emergency care coordinator (PECC) associated with performance on pediatric quality-of-care measures in emergency departments?

**Findings:**

In this cohort study of 4 645 937 visits to 858 hospitals, the presence of a PECC was differentially associated with some quality-of-care measures, such as imaging use, but not other measures, such as return visits.

**Meaning:**

These data suggest a potential influence of PECCs on processes of care, but additional work is needed to better understand the potential influence of PECCs on clinical outcomes for pediatric patients.

## Teaser

This cohort study of pediatric patients treated in emergency departments examines whether the presence of a pediatric emergency care coordinator is associated with pediatric quality-of-care measures.

## Introduction

General emergency departments (EDs) may face challenges in providing high-quality care for pediatric patients. Approximately 90% of pediatric visits occur in general EDs, and more than half of EDs care for fewer than 10 children per day, limiting exposure to the pediatric population.^1^ Most hospitals are becoming less able to provide definitive inpatient acute care to children.^2^ The loss of pediatric inpatient resources and capabilities is leading to increased regionalization of pediatric care,^3^ meaning that pediatric consultation and admission often require interhospital transfer, even for relatively straightforward conditions.^3^

Pediatric readiness, as measured by the National Pediatric Readiness Project (NPRP) assessment, is focused on ensuring that all EDs have the pediatric competencies, policies, equipment, and resources to provide high-quality emergency care to children.^4^ The NPRP checklist measuring readiness includes domains such as administration and coordination of care, including having pediatric emergency care coordinators (PECCs; staff members responsible for pediatric readiness) as well as quality improvement guidelines, ED policies and procedures focused on pediatric care, health care professional training, disaster preparedness, evidence-based guidelines for pediatrics, transfer guidelines, best practice safety guidance, and guidelines for medication, equipment, and supplies. Improved readiness is associated with decreased mortality^5,6,7^ and reduced disparities.^8^ However, pediatric readiness is not available to all, with only 55% of children living within 30 minutes of an ED with the highest levels of readiness.^9^ It is recommended that EDs have both a physician and nurse PECC, and although having a PECC is strongly associated with higher readiness,^10^ only 15% to 20% of general EDs have a PECC.^11^ The most recent national assessment of pediatric readiness demonstrated a decrease in readiness from 2013 to 2021, specifically due to decreases in administration and coordination (including PECCs).^12^

Although some factors associated with pediatric readiness are uncontrollable (eg, patient volume), modifiable factors, such as appointment of a PECC, can affect outcomes. Significant policy efforts and implementation resources have aimed to increase the number of EDs that have a PECC,^13,14^ yet the specific impact of PECCs on pediatric quality and outcomes remains undefined. The goal of this study was to examine the association between PECC presence and ED performance as reflected by pediatric quality-of-care measures.

## Methods

### Data Sources, Setting, and Participants

For this cohort study, we created an analytic cohort combining several databases, including the 2019 National ED Inventory (NEDI)–USA,^15^ 2019 Agency for Healthcare Research and Quality State Emergency Department Database (SEDD) and State Inpatient Database (SID), 2020 Supplemental NEDI-USA PECC Survey,^16^ and the 2021 NPRP Survey, for 8 geographically diverse states: Arkansas, Florida, Iowa, Maryland, Nebraska, New York, Vermont, and Wisconsin. These states were chosen because of their high-quality pediatric linkage in the SEDD and SID data. The NEDI-USA survey collects information about basic ED characteristics, including total and child visit volumes, as well as PECC status. The SEDD and SID contain data on ED discharges and inpatient admissions for each state by hospital; methods for database linkage for NEDI-USA, SEDD, and SID have been previously described^17^ and were used to link NPRP as well. The NPRP survey is a web-based assessment of ED leadership in US hospitals assessing adherence to pediatric readiness guidelines.^12,13^ The NPRP has varied rates of data missingness in states, which we addressed by using the NEDI-USA PECC variable in primary analyses and NPRP in the sensitivity analyses. The Supplemental NEDI-USA PECC Survey has been previously described^16^ and examined 4 core PECC tasks: participation in quality improvement initiatives, provision of pediatric education, verification of staff skills, and ensuring medications, equipment, supplies, and resources for children, as well as time spent on the role. We combined those core tasks as a measure of intensity of PECC implementation. This study was reviewed by the Mass General Brigham institutional review board and determined to be exempt from approval and informed consent due to the use of secondary data. The study followed the Strengthening the Reporting of Observational Studies in Epidemiology (STROBE) reporting guideline.

The following assumptions were made: (1) care delivered at freestanding children’s hospitals is distinct, and all would be expected to have someone in a PECC role; and (2) the scope of the PECC role may vary in accordance with pediatric inpatient resources at the hospital. Freestanding children’s hospitals were excluded from analysis a priori and analyses were stratified by pediatric resources among the general hospitals to account for both potential confounding by resource and the differential association with the PECC functionality from the underlying hospital capability. Freestanding children’s hospitals were defined as those that had 70% or more visits by children and/or restricted admissions to children according to American Hospital Association data. On the basis of the available pediatric resources, we categorized hospitals into 3 groups: highly pediatric resourced, moderately pediatric resourced, and non–pediatric resourced. Highly pediatric resourced hospitals were those that admitted children and had a pediatric intensive care unit (PICU). Visit thresholds to qualify for admitting children were more than 25 inpatient admissions annually. The PICU admissions were determined by more than 25 critical care admissions (critical care charges during an admission for patients aged 1-15 years) annually. The lower age cutoff was to avoid PICU determination based on neonatal intensive care unit admissions. A moderately pediatric-resourced hospital was defined as one that admitted children but did not have a PICU. A hospital without the capability to admit children and without a PICU was considered to be non–pediatric resourced. Visit volume and admissions were determined directly from the SEDD and SID. For the state where required data regarding critical care charges were not available (Vermont), we used American Hospital Association data to determine PICU capabilities. Given our a priori expectation that the PECC and other hospital factors may have a differential association depending on the underlying pediatric resources, we stratified analyses into highly, moderately, and non–pediatric-resourced hospital categories. In the adjusted analysis, we compared pediatric-resourced (high and moderate) with non–pediatric-resourced hospitals.

Our analytic cohort included all patients presenting to included EDs (full sample). We also created a second cohort (acute care sample) as a sensitivity analysis, which included only children requiring hospitalization or transfer to another hospital or who died in the ED during the index ED visit, to be consistent with prior literature evaluating pediatric readiness.^6^

### Exposures and Covariates

The primary exposure was presence of a PECC (yes or no), as defined by the NEDI-USA survey: a PECC is a physician, nurse, or advanced practice clinician who oversees administrative aspects of pediatric care in the ED that may include ensuring the presence of appropriate pediatric equipment, protocols for pediatric emergency care, pediatric education for other ED staff, and/or pediatric quality improvement activities^14^; this person is not required to have specialty pediatric training. We also examined the number of tasks completed by the PECC (Supplemental NEDI-USA survey) and hours devoted to the role. Predefined covariates at the patient level included age, sex, race and ethnicity (defined as Hispanic, non-Hispanic Black, non-Hispanic White, non-Hispanic other, and missing as captured in the Healthcare Cost and Utilization Project [HCUP] data), insurance status (defined as public, private, or other), and presence of a complex chronic condition^18^ (CCC; yes or no; ascertained from the SEDD and SID). Race of “other” included Asian or Pacific Islander, Native American, and other (per HCUP’s coding, which can include an unspecified race or multiracial, depending on the state). Race and ethnicity data were collected as a proxy for the implications of structural racism on health care access and outcomes. We calculated a modified pediatric readiness score with the PECC category (worth 19 points) removed. The total possible points were 81, and the modified score was rescaled to a range of 0 to 100, with lower scores indicating lower readiness. Predefined hospital-level covariates included pediatric ED visit volume (SID and SEDD), race and ethnicity of the patient mix (SID and SEDD), complexity of the patient mix (SID and SEDD) defined by 1 or more CCCs, modified pediatric readiness score with PECC removed (hereafter referred to as readiness score), and workplace staffing factors, including physician and nurse staffing (NPRP).

### Measures

We chose 7 pediatric quality-of-care measures that had been previously described in the literature as being amenable to use in administrative datasets,^19^ addressing process and utilization, which could be ascertained in the SID and SEDD. The 5 process measures were length of stay (LOS) longer than 1 day for patients discharged from the ED, left against medical advice or without completing treatment for patients discharged from the ED, death in the ED, return visits within 3 days, and return visits with admission within 3 days. LOS is calculated by the difference between the admission and discharge date, and so same-day stays (up to 24 hours) are coded as 0. The 2 use measures were evidence-based quality measures for reduced radiographic study use: use of chest radiography in patients with asthma among those older than 2 years and use of head computed tomography (CT) for patients with head trauma.^19,20^

Both LOS and return visit measures were unable to be run in the acute care cohort because the denominator requires an ED visit without transfer or admission, but, by definition, all patients in this cohort were transferred, admitted, or died. Similarly, left against medical advice or without completing treatment was not applicable for the acute care cohort because it would represent leaving against medical advice from an inpatient setting only.

We used a multistep process to identify radiology procedures in the SID and SEDD due to differing reporting methods by state and database. We used *International Statistical Classification of Diseases and Related Health Problems, Tenth Revision, Procedure Coding System* and *Current Procedural Terminology* codes specific to each type of imaging study, if available, along with charge or revenue codes. For states with charge codes reported, we used the radiology charge variable (eg, CHG11 for Florida) as a proxy. For states where revenue codes were reported, we used the imaging-specific codes from UB-92 or UB-04 definitions as defined by the National Uniform Billing Committee for each type of imaging study. Transfers were identified using the unique patient identifier in the SEDD and SID with the following rules: must have an index ED visit from 1 hospital, index ED visit must have a disposition of transfer, a subsequent ED or inpatient record exists with a different hospital identifier, and the gap between records must be 1 day or less.

### Statistical Analysis

Analyses were conducted from February 15, 2023, to July 9, 2024. For each quality measure, we assessed the unadjusted association with PECC presence, stratified by hospital type (pediatric-resourced or non–pediatric resourced). We constructed mixed-effects linear models to examine the association between PECC and each quality measure. The model included a random intercept for hospital, patient factors (age, sex, race and ethnicity, insurance status, and presence of a CCC), and hospital factors (PECC, pediatric ED visit volume, and race and ethnicity, insurance, and CCC of the patient mix). We hypothesized that PECCs would be associated with improved overall pediatric readiness. To evaluate the extent to which readiness mediated any PECC-quality associations, we conducted a mediation analysis. We used the medeff package in Stata, version 15.1 (StataCorp LLC) to estimate the total, direct, and average mediation effect.^21,22^

We planned several sensitivity analyses. The first examined associations excluding transfers to avoid challenges in attribution of imaging. For example, receiving hospitals are less likely to obtain images if imaging was obtained at the presenting hospital. Second, we separately analyzed a cohort limited to individuals discharged from the ED, where there will be no potential confounding by inpatient care pathways. We also examined the association between PECC presence and quality measures, adjusting for pediatric emergency medicine–trained staff and the association between intensity of PECC program implementation and quality measures for measures where an association was seen between PECC presence and quality measure performance. *P* < .05 was used to indicate statistical significance.

## Results

There were 5 119 225 ED visits by pediatric patients 18 years or younger to 1074 hospitals in the dataset during 2019, of which 216 hospitals were excluded for being either a non–acute care hospital, having no ED visits for children, or being unable to match with a NEDI-eligible ED (Figure). Patient-level characteristics for the overall sample are given in Table 1 (including freestanding children’s hospitals); across all hospital types, most children did not have a CCC and were discharged home. There were 4 645 937 visits from pediatric patients (mean [SD] age, 7.8 [6.1] years; 51% male and 49% female; 23% Hispanic, 25% non-Hispanic Black, 40% non-Hispanic White, 9% non-Hispanic other, and 3% missing race and ethnicity) to 849 non-freestanding pediatric hospitals in the analytic sample. Highly resourced pediatric centers (52 of 59 [88%]) were most likely to have a PECC compared with moderately resourced (54 of 156 [35%]) and non–pediatric-resourced hospitals (66 of 519 [13%]). Of those with a PECC, the more pediatric-resourced hospitals also reported that their PECCs were completing more of the PECC tasks and with more hours devoted to the job (Table 2). Hospitals with greater pediatric resources also reported higher readiness scores and more pediatric or pediatric emergency medicine staffing, as well as increased nurse training. Non–pediatric-resourced sites were also more likely to be lower volume, rural, and nonacademic.

**Figure.**
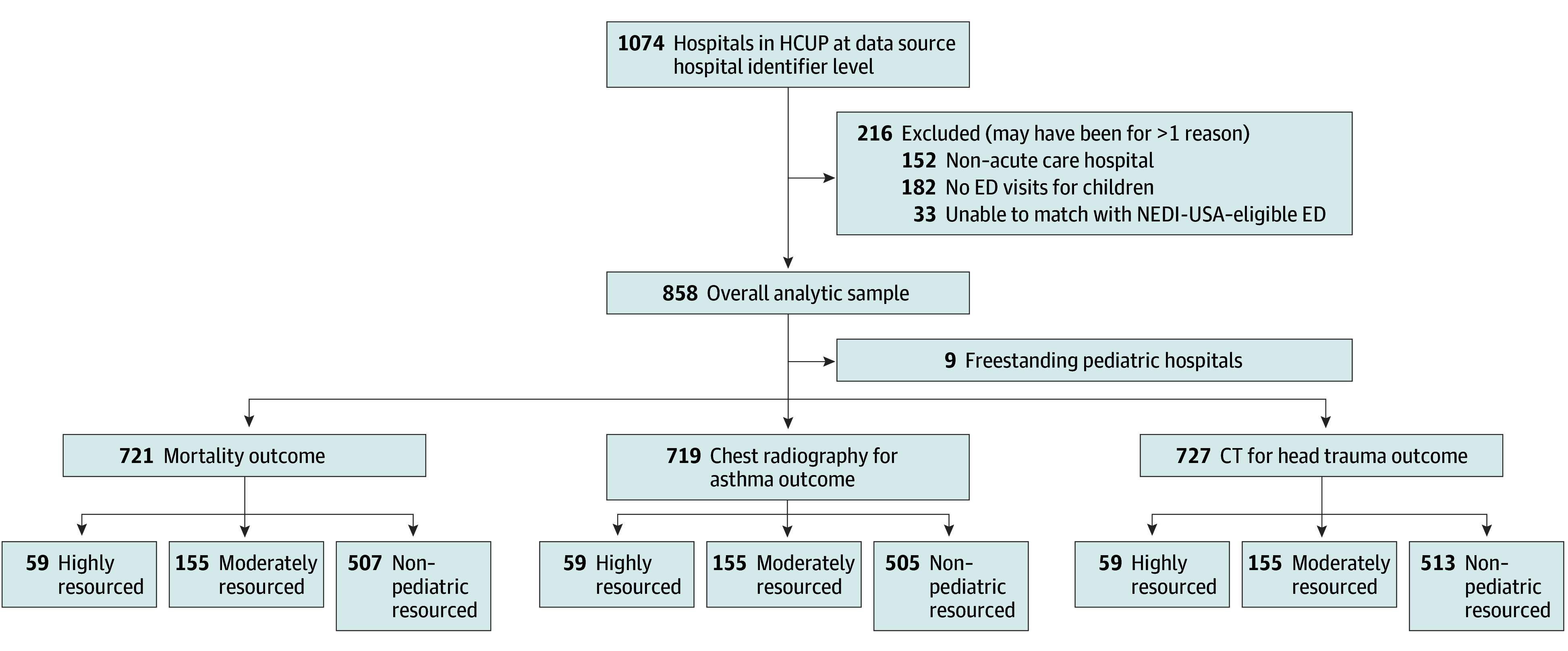
Inclusion of Hospitals in the Analytic Cohort CT indicates computed tomography; ED, emergency department; HCUP, Healthcare Cost and Utilization Project; and NEDI-USA, National Emergency Department Inventory–USA.

**Table 1.** Patient-Level Characteristics of the 5 119 225 Study Participants

Characteristic	No. (%) of patients^a^			
	Freestanding pediatric hospitals (n = 473 288 ED visits)	Highly resourced hospitals (n = 1 523 082 ED visits)	Moderately resourced hospitals (n = 1 368 248 ED visits)	Non–pediatric-resourced hospitals (n = 1 754 607 ED visits)
Age, mean (SD), y	6.0 (5.4)	6.9 (5.9)	7.8 (6.2)	8.6 (6.1)
Age, median (IQR), y	4 (1-10)	5 (2-12)	7 (2-14)	8 (3-14)
Sex				
Male	250 515 (53)	787 127 (52)	691 853 (51)	876 260 (50)
Female	222 773 (47)	735 895 (48)	676 214 (49)	878 273 (50)
Missing	0	60	181	74
Race and ethnicity				
Hispanic	147 772 (31)	448 452 (29)	296 624 (22)	344 057 (20)
Non-Hispanic Black	125 703 (27)	471 037 (31)	312 127 (23)	358 238 (20)
Non-Hispanic White	125 745 (27)	422 167 (28)	554 965 (41)	877 780 (50)
Non-Hispanic other^b^	36 456 (8)	146 932 (10)	151 894 (11)	116 398 (7)
Missing	37 612 (8)	34 494 (2)	52 638 (4)	58 124 (3)
Insurance status				
Private insurance	136 136 (29)	397 075 (26)	373 361 (27)	460 175 (26)
Public insurance	312 965 (66)	1 003 112 (66)	864 093 (63)	1 112 148 (63)
Uninsured or self-pay	14 467 (3)	88 184 (6)	86 264 (6)	121 192 (7)
Other	9666 (2)	33 847 (2)	44 391 (3)	59 671 (3)
Missing	54	864	139	1421
Zip code income quartile				
First (lowest)	195 450 (42)	611 769 (41)	471 906 (35)	589 910 (34)
Second	116 828 (25)	386 461 (26)	360 114 (27)	583 331 (34)
Third	100 221 (21)	313 150 (21)	287 701 (21)	374 543 (22)
Fourth (highest)	57 887 (12)	197 685 (13)	238 721 (18)	182 403 (11)
Missing	2902	14 017	9806	24 420
CCCs				
0	451 862 (95)	1 478 681 (97)	1 355 382 (99)	1 744 252 (99)
1	18 816 (4)	39 870 (3)	12 440 (1)	10 104 (1)
≥2	2610 (1)	4531 (0.3)	426 (0.03)	251 (0.01)
Length of stay for discharged patients, d				
Mean (SD)	0.5 (2.7)	0.4 (2.3)	0.3 (1.3)	0.1 (0.7)
Median (IQR)	0 (0-0)	0 (0-0)	0 (0-0)	0 (0-0)
Final disposition				
Discharged home	432 264 (91)	1 385 888 (91)	1 271 801 (93)	1 677 838 (96)
Admitted or transferred	38 045 (8)	120 035 (8)	71 975 (5)	48 433 (3)
Discharged to SNF, ICF, or other^c^	1797 (0.4)	8226 (0.5)	13 344 (1)	14 893 (1)
Left against medical advice	1117 (0.2)	8499 (0.6)	10 568 (0.8)	12 879 (0.7)
Died	65 (0.01)	427 (0.03)	357 (0.03)	432 (0.02)
Unknown	0	7 (<0.01)	203 (0.1)	132 (0.01)

Abbreviations: CCCs, complex chronic conditions; ED, emergency department; ICF, intermediate care facility; SNF, skilled nursing facility.

^a^ Unless otherwise indicated.

^b^ Other includes Asian or Pacific Islander, Native American, and other (per Healthcare Cost and Utilization Project coding, which can include an unspecified race or multiracial depending on the state).

^c^ Includes SNF, ICF, another type of facility that is not a short-term acute care hospital (eg, psychiatric hospital, hospice in a medical facility, long-term care hospital, custodial facility), or home health care.

**Table 2.** Hospital-Level Characteristics of the 858 Included Hospitals

Characteristic	No. (%) of hospitals^a^			
	Freestanding pediatric hospitals (n = 9)^b^	Highly resourced hospitals (n = 71)^b^	Moderately resourced hospitals (n = 179)^b^	Non–pediatric-resourced hospitals (n = 599)^b^
PECC presence^c^				
Any	9 (100)	52 (88)	54 (35)	66 (13)
Missing	0	12	23	80
PECC intensity, No. of tasks^d^^,^^e^				
1	0	1 (3)	2 (5)	2 (4)
2	0	2 (5)	3 (7)	5 (10)
3	1 (17)	5 (13)	7 (17)	15 (30)
4	5 (83)	32 (80)	29 (71)	28 (56)
Missing	3	33	139	550
PECC intensity devoted to the role, h/wk^e^				
Mean (SD)	45.7 (32.4)	32.7 (29.5)	28.1 (25.1)	15.4 (25.6)
Median (IQR)	37 (20-80)	24 (8-40)	22 (8-40)	5 (1-12)
Missing	3	33	139	550
NPRP score^f^				
Mean (SD)	96.0 (6.9)	85.1 (13.5)	76.9 (13.8)	67.4 (14.3)
Median (IQR)	98.5 (94.6-100)	90.2 (73.2-97.5)	75.0 (66.2-89.7)	65.4 (57.5-78.3)
Missing	2	20	77	264
Modified NPRP score^f^				
Mean (SD)^g^	95.0 (8.5)	82.8 (16.7)	71.9 (18.3)	60.3 (18.9)
Median (IQR)^g^	98.1 (93.4-100)	88.0 (68.7-96.9)	73.1 (57.0-87.8)	56.8 (46.8-74.8)
Missing	2	24	96	290
Physician training				
Emergency medicine	2 (33)	41 (80)	90 (90)	219 (80)
Missing	3	20	78	326
Pediatric emergency medicine	7 (100)	39 (76)	21 (21)	16 (6)
Missing	2	20	79	337
Pediatrics	6 (100)	34 (67)	26 (26)	11 (4)
Missing	3	20	80	338
Nurse training^f^				
Pediatric continuing education	7 (100)	48 (100)	93 (99)	285 (98)
Missing	2	23	85	308
Maintenance of specialty certification	6 (86)	21 (44)	21 (22)	41 (14)
Missing	2	23	85	308
Hospital-specific competencies	7 (100)	47 (98)	91 (97)	246 (85)
Missing	2	23	85	309
Visit volume, No. of visits^b^				
<1800	0	0	13 (7)	294 (49)
1800-4999	0	3 (4)	63 (35)	182 (30)
5000-9999	0	8 (11)	57 (32)	62 (10)
≥10 000	9 (100)	60 (85)	46 (26)	61 (10)
Rural^c^	0	2 (3)	33 (19)	285 (48)
Academic^c^	2 (22)	30 (43)	16 (9)	7 (1)
Council of Teaching Hospitals member^c^	4 (44)	28 (39)	13 (7)	8 (1)
Separate pediatric area^c^	9 (100)	20 (34)	58 (37)	75 (14)
Missing	0	12	23	80
Patient demographics, median (IQR)^b^				
Hispanic, %	18 (14-44)	26 (11-43)	14 (6-25)	8 (3-21)
Missing, No.	1	6	31	182
Non-Hispanic Black, %	36 (16-42)	25 (13-44)	20 (8-31)	11 (3-24)
Missing, No.	1	6	31	182
Non-Hispanic White, %	33 (23-40)	27 (12-44)	48 (25-70)	66 (40-84)
Missing, No.	1	6	31	182
Uninsured, %	73 (67-79)	78 (69-87)	80 (65-94)	70 (57-83)
Missing, No.	0	0	1	97
With ≥1 CCCs, %	5 (4-6)	3 (1-4)	1 (1-2)	0.5 (0.2-1)
Missing, No.	0	0	1	97

Abbreviations: CCCs, complex chronic conditions; NPRP, National Pediatric Readiness Project; PECC, pediatric emergency care coordinator.

^a^ Unless otherwise indicated.

^b^ Data source: 2019 State Emergency Department Database and State Inpatient Database.

^c^ Data source: 2019 National Emergency Department Inventory–USA.

^d^ Four core PECC tasks: participation in quality improvement initiatives, provision of pediatric education, verification of staff skills, and ensuring medications, equipment, supplies, and resources for children.

^e^ Data source: 2019 National Emergency Department Inventory–USA supplement.

^f^ Data source: NPRP.

^g^ Modified score has PECC presence category (worth 19 points) removed. Total possible points were 81; modified score is rescaled to a range of 0 to 100, with lower scores indicating lower readiness.

In the unadjusted models (Table 3), among highly or moderately pediatric-resourced hospitals, PECC was associated with LOS longer than 1 day among patients discharged from the ED (odds ratio [OR], 1.75; 95% CI, 1.20-2.54) and decreased odds of chest radiography in asthma (OR, 0.74; 95% CI, 0.65-0.85) and CT in head trauma (OR, 0.75; 95% CI, 0.65-0.86). In the non–pediatric-resourced hospitals, presence of a PECC was associated with decreased odds of return visit within 3 days (OR, 0.85; 95% CI, 0.76-0.95) but was not associated with return visits with admission. Presence of a PECC was also associated with decreased head CT in the non–pediatric-resourced hospitals (OR, 0.79; 95% CI, 0.69-0.90).

**Table 3.** Unadjusted Associations Between PECC Presence and Each Quality Measure^a^

Measure	Highly or moderately resourced hospitals			Non–pediatric-resourced hospitals		
	Hospitals, No.	Patients, No.	Unadjusted OR (95% CI)	Hospitals, No.	Patients, No.	Unadjusted OR (95% CI)
Process						
LOS >1 d	214	2 415 947	1.75 (1.20-2.54)^b^	514	1 483 379	1.24 (0.82-1.87)
Left against medical advice or without completing treatment	214	2 573 480	1.06 (0.77-1.45)	514	1 502 501	0.73 (0.51-1.02)
Death in ED or hospital	214	2 573 480	1.22 (0.94-1.59)	507	1 500 425	0.87 (0.62-1.22)
Return visits within 3 d	214	2 415 947	0.95 (0.85-1.07)	514	1 483 379	0.85 (0.76-0.95)^b^
Return visits with admission within 3 d	214	2 415 947	1.12 (0.91-1.37)	514	1 483 379	0.93 (0.72-1.19)
Utilization						
Chest radiography for asthma	214	218 371	0.74 (0.65-0.85)^b^	505	88 541	0.88 (0.74-1.05)
CT for head trauma	214	209 565	0.75 (0.65-0.86)^b^	513	139 603	0.79 (0.69-0.90)^b^

Abbreviations: CT, computed tomography; ED, emergency department; LOS, length of stay; OR, odds ratio; PECC, pediatric emergency care coordinator.

^a^ Shown are the odds of each measure at the patient level from a generalized linear mixed model, with random intercept for hospital and presence of any type of PECC (per 2019 National Emergency Department Inventory–USA) compared with no PECC.

^b^ Statistically significant at *P* < .05.

In the multivariable models (Table 4), among pediatric-resourced hospitals, the adjusted OR (AOR) for decreased odds of head CT in PECC hospitals was 0.85 (95% CI, 0.73-1.00). For chest radiography in asthma, PECC presence was associated with decreased odds in the pediatric-resourced hospitals (AOR, 0.77; 95% CI, 0.66-0.91). Presence of a PECC remained associated with higher rates of prolonged LOS and decreased rates of leaving against medical advice or without completing treatment among the non–pediatric-resourced hospitals. For non–pediatric-resourced hospitals, PECC presence was associated with decreased odds of CT in head trauma (AOR, 0.76; 95% CI, 0.66-0.87) but not chest radiography in asthma (AOR, 0.93; 95% CI, 0.78-1.12).

**Table 4.** Adjusted Associations Between PECC Presence and Each Quality Measure^a^

Measure	AOR (95% CI)			
	Full cohort		Subcohort	
	Highly or moderately resourced hospitals	Non–pediatric-resourced hospitals	Highly or moderately resourced hospitals	Non–pediatric-resourced hospitals
Process				
LOS >1 d^b^	1.15 (0.77-1.73)	1.56 (1.07-2.29)^c^	NA	NA
Left against medical advice or without completing treatment^d^	1.31 (0.89-1.91)	0.55 (0.40-0.77)^c^	NA	NA
Death in ED or hospital	0.95 (0.71-1.26)	0.93 (0.67-1.29)	0.93 (0.64-1.36)	0.53 (0.29-0.99)^c^
Return visits within 3 d^b^	1.02 (0.90-1.16)	0.93 (0.84-1.03)	NA	NA
Return visits with admission within 3 d^b^	1.02 (0.80-1.31)	1.08 (0.86-1.34)	NA	NA
Utilization				
CT for head trauma	0.85 (0.73-1.00)	0.76 (0.66-0.87)^c^	0.80 (0.51-1.23)	0.57 (0.24-1.35)
Chest radiography for asthma	0.77 (0.66-0.91)^c^	0.93 (0.78-1.12)	0.82 (0.51-1.31)	0.38 (0.08-1.75)

Abbreviations: AOR, adjusted odds ratio; CT, computed tomography; ED, emergency department; LOS, length of stay; NA, not applicable; PECC, pediatric emergency care coordinator.

^a^ Shown are the odds of each measure at the patient level from a generalized linear mixed model for presence of any type of PECC (per 2019 National Emergency Department Inventory–USA) compared with no PECC. Each model is run separately, with a random intercept for ED and controlling for patient age, sex, race and ethnicity, insurance status, any complex chronic condition, ED pediatric visit volume, ED quartile of non-Hispanic Black patients, ED quartile of Hispanic patients, ED quartile of patients with public insurance, and ED quartile of patients with 1 or more complex chronic condition.

^b^ Measures for LOS and return visits are not applicable to the subcohort due to the definition of the population (ie, ED admissions without admission or transfer).

^c^ Statistically significant at *P* < .05.

^d^ Measure for left against medical advice or without completing treatment not applicable for subcohort because it would represent left against medical advice from an inpatient setting only.

Mediation analysis demonstrated that the NPRP score was a significant mediator for the association between PECC presence and chest radiography for asthma in the pediatric-resourced hospitals and for the association between PECC presence and CT for head trauma in the non–pediatric hospitals (eTable 2 in Supplement 1). Mediation analyses were unable to be completed for LOS and leaving against medical advice, given the size of the dataset, and were unstable for the acute care cohort, given the small number of observations. Presence of a pediatric emergency medicine–trained physician was not significantly associated with mortality, chest radiography in asthma, or CT in head trauma in the fully adjusted model among either hospital group (eTable 3 and eTable 4 in Supplement 1).

In the sensitivity analysis examining the acute care cohort, an association between PECC presence and decreased odds of death in the ED or hospital was seen in the non–pediatric-resourced hospitals (AOR, 0.53; 95% CI, 0.29-0.99). Within the full cohort, when limiting the results to exclude transfers or those admitted to the hospital, the association between PECC presence and CT use for head trauma was significant in both hospital categories (eTable 1 in Supplement 1). Intensity of PECC program implementation was not consistently associated with the quality measure performance, whether measured by number of tasks completed (eTable 5 in Supplement 1), specific tasks completed (eTable 6 in Supplement 1), or hours spent in the role (eTable 7 in Supplement 1).

## Discussion

In this geographically diverse, 8-state cohort, prevalence and intensity of implementation of PECC programs was highest in institutions that had greater pediatric resources. However, in adjusted analyses, PECC presence was not consistently associated with higher performance on pediatric quality-of-care measures. As pediatric care is continuing to regionalize,^3^ ongoing efforts are needed to ensure that all EDs are capable of providing high-quality care for children to maintain access to high-quality emergency care.

We acknowledge that there are several potential explanations for the diverse association between PECC and quality measures in our data. Our data suggest there may be an association between PECC presence and decreased rates of CT in head trauma, both in the primary analysis and in the sensitivity analysis excluding transfers and limiting to patients discharged from the ED. This finding implies that the impact of PECC presence may be greatest where clear clinical guidelines exist, and prior literature has shown that PECC presence is associated with higher quality of resuscitation care.^23^ We hypothesize that given the widely disseminated Pediatric Emergency Care Applied Research Network (PECARN) head CT guidelines,^24^ it may be easier for PECCs to influence education and practice regarding CT use for head trauma, in contrast to guidance regarding chest radiography in pneumonia, which is less standardized and may be more challenging to implement. The interplay of the PECC role and clinical practice guidelines requires further investigation to determine whether adoption of clinical care pathways or decision support tools may play a role in improving quality of care for children in low-volume and low-resourced centers.

An additional potential contributor is that PECC program implementation is widely heterogenous.^25^ Prior work demonstrates variation in components of the PECC role implemented^16^ and that many centers without a PECC in fact have staff doing similar work but under a different job title.^25^ Given this overlap between PECC and non-PECC sites, it may be difficult to detect an association with quality. However, it is still critically important to recognize that the current state of the PECC role is insufficient to influence administrative quality measures and that, therefore, more work is needed to ensure access to high-quality pediatric emergency care for all children. Finally, PECC status may have a differential association with measures, depending on other resource availability (eg, hospital pediatric readiness) as suggested by the NPRP score serving as a moderator of the association. Although some qualitative data suggest factors that may make a PECC more effective,^25^ more work is needed on how to optimize the role across a wide variety of EDs and system resources.

Structural factors, such as pediatric facility recognition programs and PECCs, have been associated with higher pediatric readiness,^26,27^ but significant variability in participation remains. The weak and heterogeneous correlations between PECC presence and the pediatric quality measures in this study suggest that structure alone does not translate to increased measured quality. Instead, additional functional activities to improve readiness are needed, which could include quality improvement initiatives, training, collaboration, or consultation. Some evidence indicates that simulation and quality improvement initiatives have potential in this space, but efforts have been limited by ED participation, with only 17% of EDs fully completing the protocol in 1 study.^28^ Additional implementation work is needed to understand how to create effective and scalable interventions to improve pediatric readiness and care quality that will be acceptable to general EDs.

Such implementation work would benefit from improved data infrastructure as well. The most robust measurement of the quality of pediatric emergency care comes from PECARN, which studies largely high-performing academic pediatric EDs and consistently demonstrates high-quality provision of care.^29,30^ In the current study, we specifically excluded freestanding children’s hospitals, which have additional resources and infrastructure to care for pediatric patients. However, improved data collection and a registry structure similar to PECARN^31^ among general EDs would allow for more extensive quality measurement to identify hospital and system factors associated with high-quality care for children.

### Limitations

This study has several limitations. We used administrative, retrospective data and so were limited to the most temporally matched datasets, which are not from identical years. The NPRP has varied rates of data missingness, which we addressed by using the NEDI-USA PECC variable in primary analyses and the NPRP in the sensitivity analyses. PECC program implementation is heterogenous, although this likely describes the functional state of hospitals with and without PECC presence. Other processes of care in hospitals may have a significant association with quality outcomes. For example, a prior study showed that having physician and nurse PECCs was associated with improved survival for children with trauma, but this difference was associated with trauma center level.^32^ Although we have adjusted for many of these in our study, there may be unmeasured confounding by other hospital factors. Additionally, there are challenges in ascertaining quality measures from administrative data, limiting our study to those that can be calculated within the SEDD and SID. We have minimal clinical variables for severity adjustment, which we addressed by (1) excluding freestanding children’s hospitals, which presumably care for the highest percentage of complex and chronically ill children; (2) separating hospitals by pediatric resources; and (3) adjusting for CCCs. In particular, we do not have data on illness severity or triage acuity. Due to concern about ability to accurately ascertain transfers, we completed sensitivity analyses excluding transfers and limited to patients discharged from the ED with broadly similar results. Additionally, in the LOS analysis, we were unable to exclude psychiatric boarders who may have particularly long LOSs while awaiting placement.

## Conclusions

In this multistate cohort study, presence of a PECC was differentially associated with pediatric quality measures. This finding suggests that additional work is needed to ensure high-quality care for children across all types of US EDs.

## Notes

Supplement 1. **eTable 1.** Sensitivity Analyses for Associations Between PECC and Each Quality Measure **eTable 2.** Mediation Analysis **eTable 3.** Adjusted Associations Between PECC and Each Quality Measure, Including PEM Staff, Among Highly/Moderately Resourced Hospitals **eTable 4.** Adjusted Associations Between PECC and Each Quality Measure, Including PEM Staff, Among Nonpediatric Hospitals **eTable 5.** Adjusted Associations Between PECC Intensity (Number of Tasks) and Each Quality Measure **eTable 6.** Adjusted Associations Between PECC Intensity (Specific Tasks) and Each Quality Measure **eTable 7.** Adjusted Associations Between PECC Intensity (Hours Devoted to Role) and Each Quality Measure

Supplement 2.
Data Sharing Statement
