# Supplementary material for: Pediatric Emergency Care Coordinator Presence and Pediatric Care Quality Measures
Source: JAMA Netw Open. 2024 Dec 18;7(12):e2451111. doi: 10.1001/jamanetworkopen.2024.51111 (PMC11656265; doi:10.1001/jamanetworkopen.2024.51111)
Supplement: Supplement 1. — eTable 1. Sensitivity Analyses for Associations Between PECC and Each Quality Measure eTable 2. Mediation Analysis eTable 3. Adjusted Associations Between PECC and Each Quality Measure, Including PEM Staff, Among Highly/Moderately Resourced Hospitals eTable 4. Adjusted Associations Between PECC and Each Quality Measure, Including PEM Staff, Among Nonpediatric Hospitals eTable 5. Adjusted Associations Between PECC Intensity (Number of Tasks) and Each Quality Measure eTable 6. Adjusted Associations Between PECC Intensity (Specific Tasks) and Each Quality Measure eTable 7. Adjusted Associations Between PECC Intensity (Hours Devoted to Role) and Each Quality Measure [file jamanetwopen-e2451111-s001.pdf]

Supplementary Online Content

Samuels-Kalow ME, Cash RE, Michelson KA, et al. Pediatric emergency care coordinator presence and pediatric care quality measures. *JAMA Netw Open*. 2024;7(12):e2451111. doi:10.1001/jamanetworkopen.2024.51111

eTable 1. Sensitivity Analyses for Associations Between PECC and Each Quality Measure

eTable 2. Mediation Analysis

eTable 3. Adjusted Associations Between PECC and Each Quality Measure, Including PEM Staff, Among Highly/Moderately Resourced Hospitals

eTable 4. Adjusted Associations Between PECC and Each Quality Measure, Including PEM Staff, Among Nonpediatric Hospitals

eTable 5. Adjusted Associations Between PECC Intensity (Number of Tasks) and Each Quality Measure

eTable 6. Adjusted Associations Between PECC Intensity (Specific Tasks) and Each Quality Measure

eTable 7. Adjusted Associations Between PECC Intensity (Hours Devoted to Role) and Each Quality Measure

This supplementary material has been provided by the authors to give readers additional information about their work.

© 2024 Samuels-Kalow ME et al. *JAMA Network Open.*

eTable 1. Sensitivity Analyses for Associations Between PECC and Each Quality Measure

	Highly/moderately resourced hospital aOR (95% CI)	Non-pediatric hospital aOR (95% CI)
Excluding transfers		
LOS>1 day	1.15 (0.77, 1.73)	**1.56 (1.07, 2.29)**
AMA/LWCT[†]	1.31 (0.89, 1.91)	**0.55 (0.40, 0.77)**
Death in ED	0.95 (0.72, 1.26)	0.93 (0.67, 1.29)
Return visits w/i 3 days	1.02 (0.90, 1.16)	0.93 (0.84, 1.03)
Return visits with admission w/i 3 days	1.02 (0.80, 1.31)	1.08 (0.86, 1.34)
CXR in asthma	**0.77 (0.65, 0.91)**	0.94 (0.78, 1.12)
CT for head trauma	**0.85 (0.72, 0.997)**	**0.77 (0.67, 0.88)**
Limited to ED discharged		
LOS>1 day*	1.15 (0.77, 1.73)	**1.56 (1.07, 2.29)**
AMA/LWCT	1.30 (0.88, 1.90)	**0.55 (0.40, 0.77)**
Death in ED	0.92 (0.68, 1.25)	0.93 (0.67, 1.30)
Return visits w/i 3 days*	1.02 (0.90, 1.16)	0.93 (0.84, 1.03)
Return visits with admission w/i 3 days*	1.02 (0.80, 1.31)	1.08 (0.86, 1.34)
CXR in asthma	**0.78 (0.66, 0.91)**	0.94 (0.79, 1.13)
CT for head trauma	**0.85 (0.73, 0.999)**	**0.76 (0.67, 0.88)**

*Denominator excludes admissions already.

[†] Denominator excludes transfers already.

Bolded result denotes P<0.05.

© 2024 Samuels-Kalow ME et al. *JAMA Network Open.*

eTable 2. Mediation Analysis

Mediation analysis not indicated for measures with no associations found in the main models.

Modified NPRP score as mediator in association between PECC and each measure in non-pediatric hospitals.

	Unadjusted*<br>OR (95% CI)	Fully adjusted<br>OR (95% CI)
**Full cohort**		
LOS>1 day	Models unable to be run due to database size	
Average mediated effect		
Average direct effect		
Total effect		
Proportion mediated		
AMA/LWCT		
Average mediated effect		
Average direct effect		
Total effect		
Proportion mediated		
CT for head trauma		
Average mediated effect	-0.046 (-0.071, -0.021)	-0.034 (-0.057, -0.012)
Average direct effect	-0.009 (-0.021, 0.003)	-0.007 (-0.017, 0.002)
Total effect	-0.037 (-0.063, -0.009)	-0.027 (-0.049, -0.004)
Proportion mediated	**19.5 (12.6, 43.8)**	**20.7 (12.4, 55.4)**
**Acute cohort only**		
Death in ED or hospital		
Average mediated effect	-0.011 (-0.028, 0.007)	-0.013 (-0.030, 0.002)
Average direct effect	0.023 (-0.006, 0.059)	0.008 (-0.016, 0.033)
Total effect	0.012 (-0.015, 0.045)	-0.006 (-0.027, 0.017)
Proportion mediated	-57.7 (-1001.7, 1115.5)	91.7 (-1759.9, 1639.5)**

*Adjusting only for PECC and NPRP score (vs. all other covariates from full model)
**Unstable estimate due to small number of observations in this subset

Modified NPRP score as mediator in association between PECC and each measure for highly/moderately resourced pediatric hospitals.

	Unadjusted*<br>OR (95% CI)	Fully adjusted<br>OR (95% CI)
**Full cohort**		
CXR for asthma		
Average mediated effect	-0.068 (-0.104, -0.031)	-0.066 (-0.111, -0.023)
Average direct effect	-0.020 (-0.050, 0.002)	-0.017 (-0.044, 0.001)
Total effect	-0.048 (-0.093, -0.001)	-0.049 (-0.094, -0.006)
Proportion mediated	**29.3 (19.0, 64.3)**	**25.0 (14.9, 73.4)**

*Adjusting only for PECC and NPRP score (vs. all other covariates from full model)
Bolded result denotes P<0.05.

© 2024 Samuels-Kalow ME et al. *JAMA Network Open.*

eTable 3. Adjusted Associations Between PECC and Each Quality Measure, Including PEM Staff, Among Highly/Moderately Resourced Hospitals

	Mortality, aOR (95% CI)	CXR in asthma, aOR (95% CI)	CT for head trauma, aOR (95% CI)
Presence of any PECC			
No	1.00 (referent)	1.00 (referent)	1.00 (referent)
Yes	1.03 (0.73, 1.44)	0.76 (0.62, 0.94)	0.77 (0.63, 0.93)
Presence of any PEM-trained physicians			
No	1.00 (referent)	1.00 (referent)	1.00 (referent)
Yes	0.94 (0.66, 1.34)	0.92 (0.73, 1.17)	0.99 (0.8, 1.23)
Missing	Not estimated	0.79 (0.44, 1.41)	Not estimated
Patient age			
0-4 years	1.00 (referent)	1.00 (referent)	1.00 (referent)
5-9 years	0.44 (0.35, 0.54)	0.76 (0.74, 0.79)	0.99 (0.95, 1.04)
10-14 years	0.56 (0.46, 0.70)	0.82 (0.80, 0.85)	2.49 (2.40, 2.59)
15-18 years	0.90 (0.75, 1.07)	0.84 (0.82, 0.87)	5.25 (5.06, 5.45)
Patient sex			
Male	1.00 (referent)	1.00 (referent)	1.00 (referent)
Female	0.73 (0.64, 0.84)	0.94 (0.92, 0.96)	1.02 (0.99, 1.05)
Race/ethnicity			
Non-Hispanic White	1.00 (referent)	1.00 (referent)	1.00 (referent)
Non-Hispanic Black	1.26 (1.05, 1.53)	0.82 (0.79, 0.84)	0.82 (0.79, 0.85)
Hispanic	0.69 (0.55, 0.88)	0.91 (0.88, 0.94)	0.87 (0.84, 0.91)
Non-Hispanic other	1.27 (0.98, 1.64)	0.96 (0.91, 1.00)	0.94 (0.89, 0.99)
Missing	5.81 (4.12, 8.20)	1.01 (0.88, 1.16)	1.10 (0.97, 1.23)
Insurance status			
Private insurance	1.00 (referent)	1.00 (referent)	1.00 (referent)
Public insurance	0.83 (0.70, 0.99)	0.86 (0.84, 0.89)	0.80 (0.77, 0.83)
Uninsured/self-pay/other/missing	2.11 (1.70, 2.63)	0.8 0(0.76, 0.83)	0.82 (0.77, 0.86)
Any CCC			
No	1.00 (referent)	1.00 (referent)	1.00 (referent)
Yes	11.37 (9.59, 13.47)	3.76 (3.55, 3.98)	3.92 (3.41, 4.50)
Pediatric ED visit volume			
<1,800 visits	1.00 (referent)	1.00 (referent)	1.00 (referent)
1,800 – 4,999 visits	0.28 (0.11, 0.76)	0.77 (0.38, 1.56)	1.40 (0.76, 2.56)
5,000 – 9,999 visits	0.18 (0.07, 0.49)	0.79 (0.39, 1.59)	1.17 (0.64, 2.14)
≥10,000 visits	0.16 (0.06, 0.41)	0.90 (0.45, 1.79)	1.14 (0.62, 2.09)
Quartile of non-Hispanic Black patients			
1 (lowest)	1.00 (referent)	1.00 (referent)	1.00 (referent)
2	1.12 (0.71, 1.76)	0.97 (0.74, 1.25)	1.00 (0.75, 1.34)
3	0.98 (0.62, 1.54)	1.01 (0.76, 1.32)	0.86 (0.64, 1.16)
4 (highest)	1.34 (0.86, 2.09)	1.22 (0.92, 1.61)	1.31 (0.99, 1.74)
Missing	0.53 (0.30, 0.92)	1.26 (0.92, 1.72)	1.03 (0.76, 1.40)
Quartile of Hispanic patients			

© 2024 Samuels-Kalow ME et al. *JAMA Network Open.*

1 (lowest)	1.00 (referent)	1.00 (referent)	1.00 (referent)
2	0.95 (0.63, 1.44)	0.87 (0.67, 1.14)	1.08 (0.83, 1.41)
3	1.15 (0.78, 1.70)	1.17 (0.90, 1.52)	1.19 (0.92, 1.53)
4 (highest)	1.06 (0.64, 1.74)	0.93 (0.67, 1.30)	1.02 (0.74, 1.41)
Quartile of patients with public insurance			
1 (lowest)	1.00 (referent)	1.00 (referent)	1.00 (referent)
2	1.16 (0.78, 1.72)	0.84 (0.65, 1.09)	1.20 (0.96, 1.51)
3	1.28 (0.84, 1.95)	0.80 (0.62, 1.04)	1.19 (0.93, 1.52)
4 (highest)	1.23 (0.79, 1.91)	0.95 (0.73, 1.22)	1.32 (1.03, 1.70)
Quartile of patients with ≥1 CCC			
1 (lowest)	1.00 (referent)	1.00 (referent)	1.00 (referent)
2	0.90 (0.59, 1.37)	1.13 (0.92, 1.39)	0.95 (0.76, 1.19)
3	1.51 (1.00, 2.26)	1.16 (0.91, 1.49)	1.25 (0.98, 1.59)
4 (highest)	2.63 (1.69, 4.07)	0.82 (0.60, 1.13)	1.03 (0.78, 1.35)

© 2024 Samuels-Kalow ME et al. *JAMA Network Open.*

eTable 4. Adjusted Associations Between PECC and Each Quality Measure, Including PEM Staff, Among Nonpediatric Hospitals

	Mortality, aOR (95% CI)	CXR in asthma, aOR (95% CI)	CT for head trauma, aOR (95% CI)
Presence of any PECC			
No	1.00 (referent)	1.00 (referent)	1.00 (referent)
Yes	1.18 (0.75, 1.87)	0.87 (0.66, 1.15)	0.79 (0.65, 0.96)
Presence of any PEM-trained physicians			
No	1.00 (referent)	1.00 (referent)	1.00 (referent)
Yes	1.11 (0.64, 1.91)	1.28 (0.88, 1.86)	0.77 (0.58, 1.01)
Missing	Not estimated	0.92 (0.56, 1.50)	Not estimated
Patient age			
0-4 years	1.00 (referent)	1.00 (referent)	1.00 (referent)
5-9 years	0.24 (0.15, 0.38)	0.76 (0.72, 0.81)	1.22 (1.15, 1.30)
10-14 years	0.26 (0.17, 0.42)	0.76 (0.72, 0.81)	3.51 (3.31, 3.71)
15-18 years	0.46 (0.33, 0.64)	0.76 (0.72, 0.81)	7.56 (7.17, 7.97)
Patient sex			
Male	1.00 (referent)	1.00 (referent)	1.00 (referent)
Female	0.74 (0.57, 0.97)	0.87 (0.83, 0.90)	1.05 (1.01, 1.10)
Race/ethnicity			
Non-Hispanic White	1.00 (referent)	1.00 (referent)	1.00 (referent)
Non-Hispanic Black	0.92 (0.62, 1.36)	0.98 (0.93, 1.03)	0.83 (0.78, 0.88)
Hispanic	0.62 (0.40, 0.98)	1.04 (0.98, 1.10)	0.92 (0.87, 0.99)
Non-Hispanic other	0.94 (0.54, 1.63)	1.07 (0.99, 1.17)	0.94 (0.86, 1.03)
Missing	1.95 (1.05, 3.65)	0.95 (0.78, 1.16)	0.91 (0.78, 1.07)
Insurance status			
Private insurance	1.00 (referent)	1.00 (referent)	1.00 (referent)
Public insurance	0.95 (0.68, 1.35)	0.87 (0.83, 0.91)	0.84 (0.80, 0.87)
Uninsured/self-pay/other/missing	2.23 (1.49, 3.34)	0.94 (0.87, 1.01)	0.79 (0.74, 0.85)
Any CCC			
No	1.00 (referent)	1.00 (referent)	1.00 (referent)
Yes	12.78 (7.36, 22.21)	1.89 (1.55, 2.29)	1.87 (1.35, 2.58)
Pediatric ED visit volume			
<1,800 visits	1.00 (referent)	1.00 (referent)	1.00 (referent)
1,800 – 4,999 visits	0.97 (0.56, 1.70)	1.14 (0.88, 1.46)	0.88 (0.73, 1.06)
5,000 – 9,999 visits	1.02 (0.53, 1.96)	1.03 (0.73, 1.45)	0.82 (0.64, 1.05)
≥10,000 visits	0.92 (0.48, 1.75)	0.99 (0.70, 1.41)	1.13 (0.88, 1.45)
Quartile of non-Hispanic Black patients			
1 (lowest)	1.00 (referent)	1.00 (referent)	1.00 (referent)
2	1.09 (0.69, 1.73)	0.73 (0.55, 0.97)	0.95 (0.78, 1.17)
3	0.99 (0.55, 1.78)	0.97 (0.69, 1.37)	0.89 (0.69, 1.16)
4 (highest)	1.01 (0.55, 1.83)	0.88 (0.63, 1.25)	0.98 (0.78, 1.24)
Missing	1.17 (0.60, 2.27)	1.21 (0.90, 1.61)	0.89 (0.72, 1.09)
Quartile of Hispanic patients			
1 (lowest)	1.00 (referent)	1.00 (referent)	1.00 (referent)
2	1.22 (0.77, 1.94)	1.13 (0.87, 1.46)	1.03 (0.85, 1.23)

© 2024 Samuels-Kalow ME et al. *JAMA Network Open.*

3	1.32 (0.78, 2.22)	0.81 (0.59, 1.11)	1.05 (0.82, 1.36)
4 (highest)	0.88 (0.48, 1.61)	1.02 (0.71, 1.47)	0.92 (0.72, 1.17)
Quartile of patients with public insurance			
1 (lowest)	1.00 (referent)	1.00 (referent)	1.00 (referent)
2	0.86 (0.56, 1.34)	1.07 (0.85, 1.34)	1.10 (0.93, 1.29)
3	0.96 (0.57, 1.59)	1.53 (1.08, 2.17)	0.97 (0.79, 1.19)
4 (highest)	0.85 (0.48, 1.52)	0.74 (0.52, 1.06)	1.03 (0.82, 1.30)
Missing	1.34 (0.53, 3.39)	0.57 (0.32, 1.02)	0.74 (0.49, 1.13)
Quartile of patients with ≥1 CCC			
1 (lowest)	1.00 (referent)	1.00 (referent)	1.00 (referent)
2	0.90 (0.61, 1.33)	0.97 (0.78, 1.20)	0.93 (0.80, 1.08)
3	1.25 (0.77, 2.04)	1.01 (0.75, 1.36)	1.03 (0.86, 1.23)
4 (highest)	Not estimated	0.95 (0.19, 4.62)	0.59 (0.37, 0.96)

© 2024 Samuels-Kalow ME et al. *JAMA Network Open.*

eTable 5. Adjusted Associations Between PECC Intensity (Number of Tasks) and Each Quality Measure

	Highly/moderately resourced hospital, aOR (95% CI)	Non-pediatric hospital, aOR (95% CI)
LOS>1 day		
1 task	1.00 (referent)	1.00 (referent)
2 tasks	0.56 (0.11, 2.93)	0.78 (0.12, 4.90)
3 tasks	0.63 (0.15, 2.62)	0.74 (0.13, 4.13)
4 tasks	0.88 (0.27, 2.84)	0.99 (0.18, 5.51)
AMA/LWCT		
1 task	1.00 (referent)	1.00 (referent)
2 tasks	4.22 (1.20, 14.76)	0.85 (0.10, 7.30)
3 tasks	2.35 (0.78, 7.05)	1.23 (0.17, 8.97)
4 tasks	2.04 (0.82, 5.07)	1.17 (0.15, 8.94)
Death in ED		
1 task	1.00 (referent)	Not estimated
2 tasks	1.20 (0.50, 2.89)	
3 tasks	1.40 (0.59, 3.30)	
4 tasks	1.16 (0.55, 2.43)	
Return visits w/i 3 days		
1 task	1.00 (referent)	1.00 (referent)
2 tasks	0.99 (0.63, 1.56)	1.11 (0.61, 2.00)
3 tasks	0.91 (0.61, 1.36)	1.20 (0.69, 2.07)
4 tasks	0.84 (0.60, 1.17)	1.27 (0.73, 2.22)
Return visits with admission w/i 3 days		
1 task	1.00 (referent)	1.00 (referent)
2 tasks	0.45 (0.18, 1.11)	**5.23 (1.20, 22.76)**
3 tasks	0.57 (0.25, 1.27)	2.24 (0.57, 8.77)
4 tasks	0.56 (0.28, 1.11)	**5.10 (1.26, 20.66)**
CXR in asthma		
1 task	1.00 (referent)	1.00 (referent)
2 tasks	**2.40 (1.26, 4.56)**	**0.40 (0.17, 0.92)**
3 tasks	**1.89 (1.10, 3.27)**	0.60 (0.27, 1.32)
4 tasks	1.31 (0.81, 2.12)	0.60 (0.27, 1.34)
CT for head trauma		
1 task	1.00 (referent)	1.00 (referent)
2 tasks	1.32 (0.73, 2.38)	**0.37 (0.19, 0.73)**
3 tasks	1.17 (0.69, 1.96)	0.69 (0.37, 1.30)
4 tasks	1.04 (0.66, 1.63)	0.57 (0.30, 1.08)

Bolded result denotes P<0.05.

© 2024 Samuels-Kalow ME et al. *JAMA Network Open.*

eTable 6. Adjusted Associations Between PECC Intensity (Specific Tasks) and Each Quality Measure

	Highly/moderately resourced hospital aOR (95% CI)	Non-pediatric hospital aOR (95% CI)
LOS>1 day		
QI (vs. no)	0.85 (0.38, 1.93)	1.17 (0.53, 2.59)
Education provision (vs. no)	0.61 (0.19, 1.92)	0.68 (0.22, 2.14)
Skill verification (vs. no)	1.21 (0.47, 3.15)	2.25 (0.74, 6.90)
Equipment supply (vs. no)	2.35 (0.95, 5.82)	**4.71 (1.04, 21.38)**
AMA/LWCT		
QI (vs. no)	1.12 (0.59, 2.13)	0.68 (0.30, 1.54)
Education provision (vs. no)	1.06 (0.43, 2.63)	2.03 (0.61, 6.72)
Skill verification (vs. no)	1.92 (0.92, 4.01)	2.15 (0.64, 7.19)
Equipment supply (vs. no)	0.65 (0.32, 1.30)	1.49 (0.31, 7.08)
Death in ED		
QI (vs. no)	0.98 (0.63, 1.51)	0.88 (0.18, 4.24)
Education provision (vs. no)	0.65 (0.34, 1.24)	0.86 (0.14, 5.37)
Skill verification (vs. no)	0.82 (0.50, 1.34)	Not estimated
Equipment supply (vs. no)	1.24 (0.75, 2.03)	Not estimated
Return visits w/i 3 days		
QI (vs. no)	0.89 (0.71, 1.12)	1.23 (0.96, 1.58)
Education provision (vs. no)	0.83 (0.60, 1.15)	1.07 (0.76, 1.50)
Skill verification (vs. no)	0.90 (0.69, 1.17)	1.07 (0.76, 1.51)
Equipment supply (vs. no)	0.90 (0.70, 1.16)	**1.82 (1.21, 2.73)**
Return visits with admission w/i 3 days		
QI (vs. no)	0.87 (0.55, 1.37)	1.77 (1.00, 3.15)
Education provision (vs. no)	0.73 (0.37, 1.44)	2.01 (0.81, 4.95)
Skill verification (vs. no)	1.02 (0.61, 1.73)	1.87 (0.82, 4.28)
Equipment supply (vs. no)	0.77 (0.48, 1.24)	1.32 (0.49, 3.55)
CXR in asthma		
QI (vs. no)	0.90 (0.64, 1.27)	1.09 (0.76, 1.55)
Education provision (vs. no)	0.99 (0.56, 1.75)	**2.25 (1.43, 3.51)**
Skill verification (vs. no)	0.95 (0.63, 1.45)	1.39 (0.84, 2.33)
Equipment supply (vs. no)	0.74 (0.53, 1.05)	0.86 (0.48, 1.54)
CT for head trauma		
QI (vs. no)	0.80 (0.58, 1.11)	0.94 (0.66, 1.34)
Education provision (vs. no)	0.76 (0.48, 1.18)	1.11 (0.69, 1.79)
Skill verification (vs. no)	0.99 (0.69, 1.41)	1.12 (0.69, 1.84)
Equipment supply (vs. no)	1.13 (0.80, 1.58)	**2.71 (1.53, 4.79)**

Note: Each is run in a separate model.
Bolded result denotes P<0.05.

© 2024 Samuels-Kalow ME et al. *JAMA Network Open.*

eTable 7. Adjusted Associations Between PECC Intensity (Hours Devoted to Role) and Each Quality Measure

	Highly/moderately resourced hospital<br>aOR (95% CI)	Non-pediatric hospital<br>aOR (95% CI)
LOS>1 day		
Per 5-hour increase	1.00 (0.94, 1.06)	0.97 (0.90, 1.05)
AMA/LWCT[†]		
Per 5-hour increase	**1.06 (1.02, 1.11)**	**0.92 (0.87, 0.99)**
Death in ED		
Per 5-hour increase	0.99 (0.97, 1.02)	0.93 (0.82, 1.05)
Return visits w/i 3 days		
Per 5-hour increase	1.00 (0.99, 1.02)	1.00 (0.98, 1.03)
Return visits with admission w/i 3 days		
Per 5-hour increase	**1.04 (1.01, 1.07)**	1.01 (0.97, 1.06)
CXR in asthma		
Per 5-hour increase	**1.03 (1.00, 1.05)**	0.99 (0.97, 1.02)
CT for head trauma		
Per 5-hour increase	1.01 (0.99, 1.03)	0.98 (0.95, 1.01)

Bolded result denotes P<0.05.

© 2024 Samuels-Kalow ME et al. *JAMA Network Open.*
