# Supplementary material for: Pediatric Emergency Care Coordinator Presence and Pediatric Care Quality Measures
Source: JAMA Netw Open. 2024 Dec 18;7(12):e2451111. doi: 10.1001/jamanetworkopen.2024.51111 (PMC11656265; doi:10.1001/jamanetworkopen.2024.51111)
Supplement: Supplement 2. — Data Sharing Statement [file jamanetwopen-e2451111-s002.pdf]

Data Sharing Statement

Samuels-Kalow. Pediatric Emergency Care Coordinator Presence and Pediatric Care Quality Measures. *JAMA Netw Open*. Published December 18, 2024. doi:10.1001/jamanetworkopen.2024.51111

Data
Data available: No
